# Antibody microarray analysis of the amniotic fluid proteome for predicting the outcome of rescue cerclage in patients with cervical insufficiency

**DOI:** 10.1042/BSR20210174

**Published:** 2021-07-02

**Authors:** Subeen Hong, Kyo Hoon Park, Young Eun Lee, Sue Shin, Hyeon Ji Kim, Yu Mi Kim

**Affiliations:** 1 Department of Obstetrics and Gynecology, College of Medicine, The Catholic University of Korea, Seoul, Korea; 2 Department of Obstetrics and Gynecology, Seoul National University College of Medicine, Seoul National University Bundang Hospital, Seongnam, Korea; 3 Department of Laboratory Medicine, Seoul National University Boramae Hospital, Seoul, Korea

**Keywords:** amniotic fluid, antibody microarray, biomarkers, cervical insufficiency, rescue cerclage, spontaneous preterm delivery

## Abstract

Little is known about the biomarkers that can identify patient candidates suitable for rescue cerclage procedure. The purpose of the study was to identify novel biomarkers in amniotic fluid (AF) that can predict the outcome of rescue cerclage in patients with cervical insufficiency by using an antibody microarray. This case–control study was conducted using AF samples collected from singleton pregnant women who underwent rescue cerclage following a diagnosis of cervical insufficiency (19–25 weeks). Patients were divided into case (*n*=20) and control (*n*=20) groups based on the occurrence of spontaneous preterm delivery (SPTD) at <34 weeks of gestation after cerclage placement. The AF proteomes were analyzed using an antibody microarray for biomarker discovery work. Ten candidate biomarkers of interest were validated by enzyme-linked immunosorbent assay (ELISA). Thirty-one molecules studied showed significant intergroup differences (≥two-fold change in signal intensity). Validation by ELISA confirmed significantly higher levels of a proliferation-inducing ligand (APRIL), S100 calcium-binding protein A8/A9 complex (S100 A8/A9), tissue inhibitors of metalloproteinase-1 (TIMP-1), macrophage inflammatory protein-1α (MIP-1α), and interleukin-8 (IL-8) in women who had SPTD at <34 weeks. Of these, AF S100 A8/A9 and TIMP-1 levels were independent of other potentially confounding factors (e.g., cervical dilatation). S100 A8/A9 had the highest area under the curve (AUC) at 0.857. Using protein–antibody microarray technology, we identified differentially expressed proteins (DEPs) and several novel biomarkers (APRIL, IL-8, MIP-1α, S100 A8/A9, and TIMP-1) in AF from women who had SPTB at <34 weeks after cerclage for cervical insufficiency. These data can provide an insight into the molecular mechanisms underlying SPTD after rescue cerclage in patients with cervical insufficiency.

## Introduction

Acute cervical insufficiency is defined as painless cervical dilatation in the second trimester and occurs at an incidence of 4.6 per 1000 births [1,2]. Although it is uncommon in the general obstetric population, it remains the main cause of early spontaneous preterm delivery (SPTD) or second-trimester abortion [3]. Rescue (or emergency) cerclage in patients with cervical insufficiency has resulted in markedly improved neonatal outcomes, with neonatal survival rates of approximately 71% [4], which supports its extensive application in clinical practice. However, despite the clinical importance of rescue cerclage and knowledge about its associated complications (membrane rupture, bleeding, and clinical chorioamnionitis), information on the biomarkers that can identify patient candidates suitable for rescue cerclage procedure remains limited.

Several studies have shown that elevated levels of pro-inflammatory cytokines and chemokines and hemoglobin peaks (for decidual hemorrhage) in the amniotic fluid (AF) are associated with poor outcomes for rescue cerclage for cervical insufficiency [5–8]. However, the clinical utility of these biomarkers remains limited with respect to prognostic assessment and treatment guidance because (i) their diagnostic sensitivity, specificity, and cut-off values have not been determined, and (ii) it is uncertain whether they are affected by confounding factors such as cervical dilation [5,6,8]. Moreover, these studies have restricted their analysis to specific target markers that are generated in response to inflammation or infection, thereby highlighting the need to explore all factors underlying mechanisms of SPTD occurring after cerclage placement in cervical insufficiency. This has prompted us to screen for novel additional biomarkers in studies with larger sample sizes to further refine prediction of the outcome of rescue cerclage for cervical insufficiency.

The membrane-based protein array, which is known to be a powerful platform for simultaneously detecting the expression levels of 507 human target proteins, has been proposed as a clinically useful method to identify disease biomarkers for diagnosis, prognosis, prediction, and understanding of previously unknown mechanisms involved in certain diseases [9–11]. However, to date, no study has used protein–antibody array technology to identify biomarkers for pregnancy outcome after placement of rescue cerclage for acute cervical insufficiency. The purpose of the study was to identify novel biomarkers in AF using an antibody microarray that can predict the outcome of rescue cerclage in patients with cervical insufficiency.

## Materials and methods

### Study design

This retrospective study was approved by the ethics committee at Seoul National University Bundang Hospital, Seongnamsi, Republic of Korea (IRB no. B-1311/228-010). All patients provided written informed consent for the amniocentesis procedure and collection and use of AF samples for research purposes. The study cohort consisted of women with a singleton pregnancy at 19–25 weeks of gestation, who underwent rescue cerclage following diagnosis of cervical insufficiency, at the Seoul National University Bundang Hospital. A case–control design was used for the present study. The inclusion criteria for case and control subjects were a live fetus, amniocentesis conducted prior to cerclage placement to evaluate AF for infection/inflammation and/or to reduce tension in the amniotic cavity and promote retraction, intact amniotic membranes, and an aliquot of AF specimen available. Women were excluded if they had multiple pregnancies, major congenital anomalies, clinical chorioamnionitis at presentation, and prophylactic cerclage during early pregnancy. A detailed database of all obstetric women admitted to the high-risk pregnancy unit for either fetal or maternal indications has been maintained at Seoul National University Bundang Hospital since 2003. Between September 2004 and December 2015, 60 women diagnosed with cervical insufficiency and also satisfying the aforementioned criteria were identified. Of these 60 patients with a singleton pregnancy, 43 patients underwent rescue cerclage for cervical insufficiency. Further, of these 43 patients, 20 patients had SPTD at <34 weeks of gestation, and 23 patients delivered at ≥34 weeks after cerclage placement. Consequently, case group patients with SPTD at <34 weeks of gestation (n=20) and the control group (n=20) matched by gestational age at sampling, maternal age, parity, and year of sampling, were selected from the 43 patients. The clinical data (but not the laboratory data measured in the AF samples) of several patients included in this manuscript have been previously published elsewhere [7,12]. The primary outcome measure was SPTD at <34 weeks of gestation after cerclage placement.

### Definition, diagnosis, and management of cervical insufficiency

Cervical insufficiency is defined as a painless cervical dilatation (≥1 cm) and exposed/bulging fetal membranes in the absence of uterine contractions as determined via visual assessment using a sterile speculum. The method for rescue cerclage placement and the associated medication have been described in detail in our previous study [7]. In brief, McDonald technique-based rescue cerclage procedures were performed on patients with cervical insufficiency. Amnioreduction was performed on patients with bulging prolapsed membranes into the vagina to reduce tension on the membranes and thereby allow for retraction before placement of the cerclage. Clinical decisions regarding cerclage placement, and tocolytic and antibiotic treatments were primarily made at the discretion of the attending physicians. Antenatal corticosteroids were administered for lung maturity in women between 24 and 34 weeks of gestation. Clinical chorioamnionitis was defined based on the criteria of Gibbs et al., as previously reported [13].

### AF sample collection and processing

Before rescue cerclage placement, transabdominal amniocentesis was performed with a 22-gauge spinal needle under sonographic guidance and aseptic conditions. AF samples were retrieved prior to the administration of medication (such as tocolytics, corticosteroids, and antibiotics). Following previously described methods [14], the samples of AF were cultured for aerobic/anaerobic bacteria and genital mycoplasmas (e.g. *Mycoplasma hominis* and *Ureaplasma urealyticum*). The leftover AF samples were centrifuged at 1500×***g*** for 10 min, aliquoted in 1.5-ml polypropylene tubes, and stored at −80°C until the analyses.

### Membrane-based human antibody array

AF proteome profiles were compared between case and control groups using protein–antibody microarray experiments. An equal amount of protein from individual AF samples (25.0 µg) was pooled into the clinical success control group (n=20) and clinical failure case group (n=20). In these pooled samples, the relative expression levels of immunoregulatory proteins were profiled using a human antibody array kit (AAH-BLM-1B-2; RayBiotech, Norcross, GA). This human antibody array kit can simultaneously detect 507 human cytokines and related proteins. AF samples from 20 women in each group (500 µg per group) were mixed and assayed in duplicate, according to the manufacturer’s protocol. The methods used for the antibody array have been previously described [11,15]. The signal intensity of each spot was normalized as a percentage of the positive controls on each membrane. Target proteins were identified when the spots (proteins) were visible to the naked eye, and simultaneously displayed ≥two-fold difference in signal intensity between clinical success and failure groups upon chemiluminescence image analysis.

### Enzyme-linked immunosorbent assay validation

Based on the antibody microarray results, we quantified the concentration of ten selected protein targets in all 40 samples. The molecules were selected on the basis of their biological functions, potential clinical relevance, magnitude of the fold change in expression, enzyme-linked immunosorbent assay (ELISA) kit availability, and little or no information on their expression change in AF associated with the poor outcome after rescue cerclage. The levels of angiopoietin-2, a proliferation-inducing ligand (APRIL), endostatin, interleukin-8 (IL-8), macrophage inflammatory protein-1α (MIP-1α), preadipocyte factor 1 (Pref-1), S100 calcium-binding protein A8/A9 complex (S100 A8/A9), thrombospondin-2, and tissue inhibitors of metalloproteinase-1 (TIMP-1), and angiostatin (RayBiotech, Norcross, GA) were determined using ELISA kits (DuoSet ELISA from R&D Systems, Minneapolis, MN, U.S.A.). The concentration of angiostatin was determined by another ELISA kit from RayBiotech, Norcross, GA. ELISAs were performed according to the manufacturer’s instructions. The ranges of the angiopoietin-2, angiostatin, APRIL, endostatin, IL-8, MIP-1α, Pref-1, S100 A8/A9, thrombospondin-2, and TIMP-1 standard curves were 93.8–6000 pg/ml, 31.2–2000 ng/ml, 31.2–2000 pg/ml, 62.50–4000 pg/ml, 31.20–2000 pg/ml, 7.81–500 pg/ml, 93.8–6000 pg/ml, 93.8–6000 pg/ml, 10–0.156 ng/ml, and 31.2–2000 pg/ml, respectively. Prior to measurement of these proteins, the AF samples were diluted at 1:4 for angiopoietin-2 and APRIL, 1:10 for angiostatin, IL-8 and MIP-1α, 1:100 for endostatin, Pref-1 and thrombospondin-2, and 1:500 for S100 A8/A9 and TIMP-1. All samples were assayed in duplicate. The intra-assay coefficient of variation (CV) was <10% for all analyzed proteins. The inter-assay CV is not applicable in these ELISA experiments, because only one ELISA plate for each protein was used in the present6 study.

### Statistical analyses

Comparison of clinical data and candidate biomarkers was performed using the Mann–Whitney U test for non-parametric variables, and comparison of categorical data using the Chi-squared test or Fisher’s exact test. Firth’s logistic regression due to the small sample size was further used to examine the independent relationship of candidate biomarker concentrations in the AF with the occurrence of SPTD at <34 weeks after cerclage, after controlling for baseline clinical variable (i.e., cervical dilatation and corticosteroid administration) with a P- value <0.05 during univariate analysis. Receiver operating characteristic (ROC) curves and the areas under the ROC curves (AUCs) were computed for each selected candidate protein for prediction of SPTD at <34 weeks of gestation after cerclage placement. The best cut-off points were determined using the maximum Youden index (maximum [sensitivity + specificity − 1]) and the AUCs were compared for each protein as previously described [16]. A value of two-tailed P<0.05 was considered statistically significant. The statistical analyses of data were carried out using SPSS version 25.0 (IBM SPSS Inc., Chicago, IL).

## Results

### Characteristics of the cohort recruited for antibody microarray analysis

Table 1 describes the baseline demographic and clinical characteristics of the cohort used for antibody microarray analysis. All women who participated in the present study were of Korean ethnicity. The cohort consisted of women with SPTD delivered at <34 weeks of gestation, and near-term controls delivered at ≥34 weeks of gestation. There were no significant differences between the two groups with regard to the gestational age at sampling, maternal age, body mass index, and parity, as a result of matching. However, women with SPTD who delivered at <34 weeks exhibited significantly more advanced cervical dilatation and were administered with a higher rate of corticosteroid than those who delivered at ≥34 weeks.

**Table 1:** Demographic and clinical characteristics of the study population recruited for antibody microarray analysis

Characteristics	Delivery < 34 weeks (*n*=20)	Delivery ≥ 34 weeks (*n*=20)	*P*-values
Age (years)	31.5 ± 2.3	32.9 ± 3.8	0.183
Body mass index (kg/m^2^)	23.3 ± 4.2	24.2 ± 3.4	0.351
Nulliparity	60.0% (12)	35.0% (7)	0.113
Gestational age at sampling (weeks)	21.8 ± 1.4	21.6 ± 1.5	0.393
Cervical dilatation	3.0 (1.5–6.0)	2.0 (1.0–8.0)	0.008
>2 cm	14 (70.0%)	5 (25.0%)	
≤2 cm	6 (30.0%)	15 (75.0%)	
Positive AF cultures	15.0% (3)	0.0% (0)	0.231
Use of tocolytics	70.0% (14)	45.0% (9)	0.110
Use of corticosteroids	45.0% (9)	5.0% (1)	0.008
Use of antibiotics	100.0% (20)	100.0% (20)	
Gestational age at delivery (weeks)	24.8 ± 3.3	37.4 ± 1.7	<0.001
Clinical chorioamnionitis	15.0% (3)	0.0% (0)	0.231

Values are given as the mean ± standard deviation, median (range) or % (*n*).

### Antibody microarray analysis

**Figure 1:**
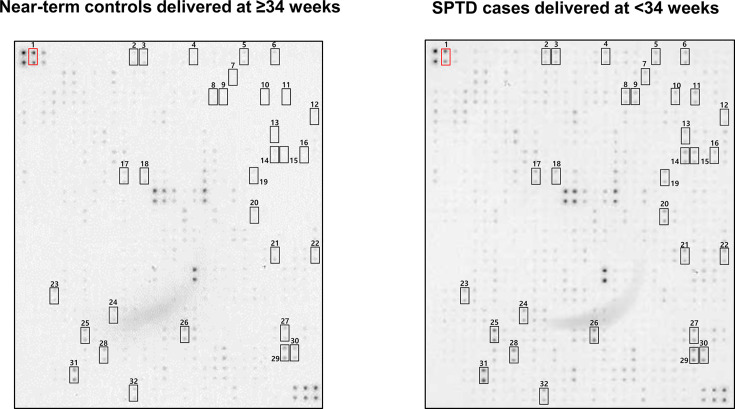
Expression levels of 507 immunoregulatory proteins in the AF of patients with cervical insufficiency who had subsequent SPTD at <34 weeks of gestation versus women who delivered at ≥34 weeks after rescue cerclage for cervical insufficiency Pooled AF samples from each group (20 women with SPTD and 20 gestational age-matched women with near-term delivery) were assayed using a human antibody array kit (AAH-BLM-1B-2; RayBiotech, Norcross, GA). Using the criteria of visibility to the naked eye and a ≥two-fold change in signal intensity, 31 proteins differentially expressed between the AF from women who had subsequent SPTD at <34 weeks of gestation after cerclage placement and AF from women who delivered at ≥34 weeks are indicated in rectangles. Number 1 shows the positive controls.

**Figure 2:**
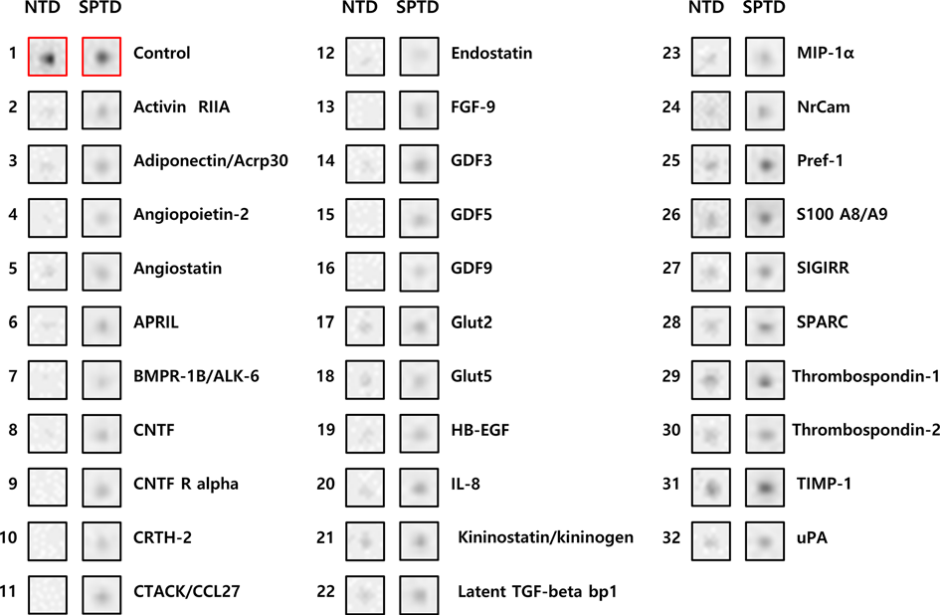
Proteins with significantly different expression between the AF from women with cervical insufficiency, who had subsequent SPTD < 34 weeks after cerclage placement and AF from women with near-term delivery (NTD), using the criteria of visibility to the naked eye and a ≥two-fold change in signal intensity

Figure 1 shows the results of protein–antibody microarray experiments on the pooled AF samples from the clinical success group versus clinical failure group in patients with cervical insufficiency treated with rescue cerclage. Using the spot selection criteria previously described in the ‘Materials and methods’ section, 31 of 507 human proteins included in the membrane-based microarray exhibited significant intergroup differences; all 31 proteins were up-regulated in AF samples from women who had SPTD at <34 weeks compared with that in the women who delivered at ≥34 weeks (Figure 2 and Table 2).

**Table 2:** Thirty-one proteins that were differentially up-regulated in AF samples from women with SPTD at <34 weeks relative to those from women who delivered at ≥34 weeks after rescue cerclage for cervical insufficiency

Identification of rectangles in Figure 1	Short name	Protein name	Fold-change
1		Positive control	
2	Activin RIIA	Activin receptor IIA	3.7
3	Adiponectin/Acrp30	Adiponectin/Acrp30	4.0
4	Angiopoietin-2	Angiopoietin-2	4.2
5	Angiostatin	Angiostatin	4.9
6	APRIL	A proliferation-inducing ligand	6.7
7	BMPR-1B/ALK-6	Bone morphogenetic protein receptor type 1B	3.9
8	CNTF	Ciliary neurotrophic factor	3.4
9	CNTF R alpha	Ciliary neurotrophic factor receptor α	3.4
10	CRTH-2	Chemoattractant receptor-homologous molecule expressed on Th2 cells	3.5
11	CTACK/CCL27	C–C motif chemokine ligand 27	4.2
12	Endostatin	Endostatin	2.2
13	FGF-9	Fibroblast growth factor 9	4.4
14	GDF3	Growth differentiation factor 3	5.5
15	GDF5	Growth differentiation factor 5	4.0
16	GDF9	Growth differentiation factor 9	3.7
17	Glut2	Glucose transporter 2	2.0
18	Glut5	Glucose transporter 5	2.1
19	HB-EGF	Heparin binding EGF-like growth factor	3.4
20	IL-8	Interleukin 8	4.2
21	Kininostatin/kininogen	Kininostatin/kininogen	2.8
22	Latent TGF-β bp1	Latent transforming growth factor β-binding protein 1	2.8
23	MIP-1α	Macrophage inflammatory protein 1-α	3.2
24	NrCam	Neuronal cell adhesion molecule	2.0
25	Pref-1	Preadipocyte factor 1	3.0
26	S100 A8/A9	S100 calcium binding protein A8/A9 complex	3.8
27	SIGIRR	Single lg IL-1 related protein	2.3
28	SPARC	Secreted protein acidic and rich in cysteine	2.0
29	Thrombospondin-1	Thrombospondin-1	2.0
30	Thrombospondin-2	Thrombospondin-2	2.6
31	TIMP-1	Tissue inhibitors of metalloproteinase 1	2.3
32	uPA	Urokinase-type plasminogen activator	3.5

### ELISA validation of selected candidate biomarkers

To validate findings from the antibody microarray, we performed a quantitative ELISA on ten selected candidate proteins associated with SPTD at <34 weeks of gestation after cerclage, namely, angiopoietin-2, angiostatin, APRIL, endostatin, IL-8, MIP-1α, Pref-1, S100 A8/A9, thrombospondin-2, and TIMP-1. The median AF levels of APRIL, IL-8, MIP-1α, S100 A8/A9, and TIMP-1 were observed to be significantly higher in women who had SPTD at <34 weeks than in women who delivered at ≥34 weeks (Table 3). The multivariable Firth’s logistic regression analysis revealed that the elevated AF levels of S100 A8/A9 and TIMP-1, but not APRIL, IL-8, and MIP-1α, were significantly associated with SPTD at <34 weeks of gestation after cerclage, when adjusted for cervical dilatation and corticosteroid administration (Table 4). However, the AF levels of angiopoietin-2, angiostatin, endostatin, Pref-1, and thrombospondin-2 did not significantly differ between the two groups (Table 3).

**Table 3:** Ten selected candidate biomarkers of interest in AF from women with cervical insufficiency, stratified according to SPTD at <34 weeks of gestation after cerclage placement

Characteristics	Delivery < 34 weeks (*n*=20)	Delivery ≥ 34 weeks (*n*=20)	*P*-values
AF angiopoietin-2 (ng/l)	7.01 ± 2.94	6.72 ± 3.47	0.607
AF angiostatin (ng/ml)	718.38 ± 302.38	719.15 ± 316.84	0.787
AF APRIL (ng/ml)	1.03 ± 0.72	0.57 ± 0.29	0.006
AF endostatin (ng/ml)	59.38 ± 12.90	57.96 ± 17.43	0.646
AF IL-8 (ng/ml)	17.20 ±31.40	4.49 ± 4.99	0.020
AF Pref-1 (ng/ml)	434.85 ± 93.52	411.56 ± 57.09	0.245
AF S100 A8/A9 (μg/ml)	5.13 ± 3.82	1.61 ± 1.46	<0.001
AF MIP-1α (ng/ml)	0.99 ± 1.49	0.33 ± 0.51	0.028
AF thrombospondin-2 (ng/ml)	12.59 ± 4.92	11.33 ± 5.08	0.512
AF TIMP-1 (μg/ml)	1.46 ± 0.61	0.94 ± 0.32	0.001

Values are given as the mean ± standard deviation.

**Table 4:** Multivariate logistic regression model showing the adjusted odds ratios of association between various proteins in AF and SPTD at <34 weeks of gestation after cerclage placement

Variables	Adjusted odds ratio^1^	95% CI	*P*-value^2^
AF APRIL (ng/ml)	3.95	0.92–62.18	0.069
AF IL-8 (ng/ml)	1.06	0.98–1.23	0.164
AF MIP-1α (ng/ml)	1.84	0.99–7.62	0.053
AF S100 A8/A9 (μg/ml)	1.95	1.17–4.31	0.002
AF TIMP -1 (μg/ml)	1.001	1.000–1.004	0.025

1 Adjustment for cervical dilation and corticosteroid administration.

2 For the adjusted odds ratio.

**Figure 3:**
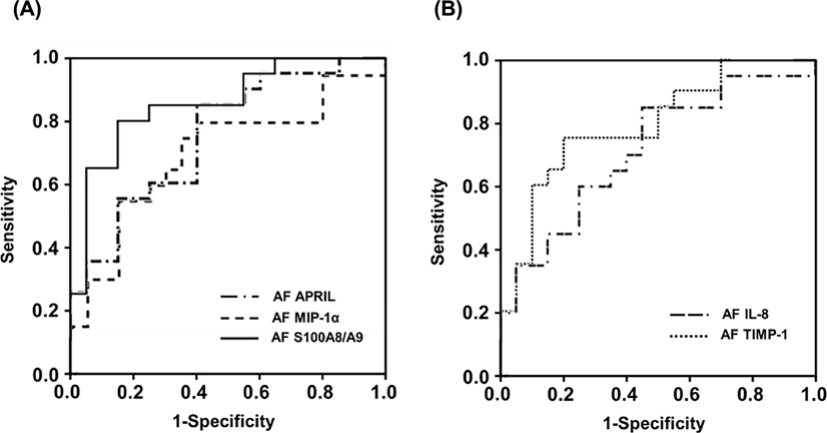
ROC curves for prediction of SPTD at <34 weeks of gestation after emergency cerclage (**A**) By APRIL, MIP-1α, and S100 A8/A9 levels in AF (AF APRIL: AUC 0.752, SE 0.077, *P*=0.006; AF MIP-1α: AUC 0.702, SE 0.085, *P*=0.028; AF S100 A8/A9: AUC 0.857, SE 0.060, *P*<0.001). (**B**) By AF IL-8 and TIMP-1 levels (AF IL-8: AUC 0.715, SE 0.082, *P*=0.020; AF TIMP-1: AUC 0.792, SE 0.072, *P*=0.002). Differences in the AUCs of AF APRIL, IL-8, MIP-1α, S100 A8/A9, and TIMP-1 were not significant (all variables: *P*=0.052–0.787) except for the AUC of AF S100 A8/A9, which was significantly greater than that of AF MIP-1α (*P=*0.023).

The AUC values of AF APRIL, IL-8, MIP-1α, S100 A8/A9, and TIMP-1 for the prediction of SPTD at <34 weeks of gestation after cerclage were 0.752, 0.715, 0.702, 0.857, and 0.792, respectively (Table 5 and Figure 3). The best cut-off values (sensitivity and specificity) for predicting SPTD at <34 weeks of gestation after cerclage were 0.502 ng/ml for AF APRIL (85% sensitivity and 60% specificity), 2.996 ng/ml for AF IL-8 (85% sensitivity and 55% specificity), 0.170 ng/ml for AF MIP-1α (80% sensitivity and 60% specificity), 2.548 μg/ml for AF S100 A8/A9 (80% sensitivity and 85% specificity), and 1.003 μg/ml for AF TIMP-1 (75% sensitivity and 80% specificity) (Table 5). The AUCs of AF APRIL, IL-8, MIP-1α, S100 A8/A9, and TIMP-1 were not significantly different (all variables: P=0.052–0.787), except for the AUC of AF S100 A8/A9, which was significantly greater than that of AF MIP-1α (P=0.023).

**Table 5:** Diagnostic indices of APRIL, MIP-1α, S100 A8/A9, and TIMP-1 in AF to predict SPTD at <34 weeks of gestation after cerclage placement

Variables	Area (± SE) under the ROC curve	95% CI	Cut-off value^1^	Sensitivity^2^ (95% CI)	Specificity^2^ (95% CI)	PPV	NPV
AF APRIL (ng/ml)	0.752 ± 0.077	0.602–0.903	≥0.502	85.0 (62.1–96.8)	60.0 (36.1–80.9)	68.0	80.0
AF IL-8 (ng/ml)	0.715 ± 0.082	0.554–0.876	≥2.996	85.0 (62.1–96.8)	55.0 (31.5–76.9)	65.4	78.6
AF MIP-1α (ng/ml)	0.702 ± 0.085	0.535–0.870	≥0.170	80.0 (56.3–94.3)	60.0 (36.1–80.9)	66.7	75.0
AF S100 A8/A9 (μg/ml)	0.857 ± 0.060^3^	0.740–0.975	≥2.548	80.0 (56.3–94.3)	85.0 (62.1–96.8)	84.2	81.0
AF TIMP -1 (μg/ml)	0.792 ± 0.072	0.652–0.933	≥1.003	75.00 (50.9–91.3)	80.0 (56.3–94.3)	79.0	76.2

Abbreviations: CI, confidence interval; NPV, negative predictive value; PPV, positive predictive value; SE, standard error.

1 Cut-off values corresponding to the highest sum of sensitivity and specificity

2 Values are presented as % (95% CI).

3 *P*<0.05 compared with AF MIP-1 α by the method proposed by DeLong et al.

## Discussion

The principal findings of the study were as follows: (i) using protein–antibody microarray analysis on pooled samples, we characterized 31 differentially expressed proteins (DEPs) in AF from women who had SPTD at <34 weeks of gestation after rescue cerclage for cervical insufficiency; (ii) validation of selected targets (ten DEPs) confirmed significant up-regulation of AF S100 A8/A9, TIMP-1, APRIL, IL-8, and MIP-1α, the first two of which were independent of other potentially confounding factors (e.g., cervical dilatation at presentation), in women with SPTD at <34 weeksf compared with women delivering at ≥34 weeks after rescue cerclage. These findings may provide insights into the molecular mechanisms of SPTD after rescue cerclage for cervical insufficiency, potential targets for the development of novel therapeutics, and identification of women candidates for the rescue cerclage procedure.

In women with cervical insufficiency, subclinical intra-amniotic infection/inflammation has been consistently reported as a prognostic factor for outcome of rescue cerclage [7,12,17,18]. This is in agreement with the current study wherein elevated levels of inflammatory markers (IL-8, MIP-1α, and S100 A8/A9) in AF are observed. Moreover, extensive research has been conducted to study the effects of systemic and cervical subclinical inflammation on the outcome of rescue cerclage for cervical insufficiency. It was found that elevated cervical mucus IL-8 level and pre-operative neutrophil-lymphocyte ratio in maternal blood are risk factors for SPTD after cerclage [7,19]. Collectively, these observations suggest that inflammatory response plays a major role in initiation and progression of preterm birth after cerclage, regardless of local (AF or cervical compartment) or systemic subclinical presentations, and that an inflammation-related approach should be included in the therapeutic strategy to improve the outcome of rescue cerclage. In fact, a randomized controlled trial has shown that addition of perioperative indomethacin and antibiotics prolongs gestational latency after examination-indicated cerclage placement [20].

We comprehensively evaluated AF proteins associated with SPTD after cerclage using a protein microarray technique, which enabled us to simultaneously profile multiple proteins in a high-throughput manner. The main advantages of this protein microarray technology over other traditional proteomic technologies (mass spectrometry) are: (1) the identities of the measured proteins are already known or characterized, providing biological interpretation of the results, (2) higher format versatility and reproducibility, (3) high sensitivity, (4) low cost, and (5) requirement of small quantities of samples. All these features have encouraged the increased use of the protein microarray kit in obstetric research; however, it is seldom used in the obstetric field. To the best of our knowledge, this is the first study to assess AF proteins in patients with cervical insufficiency using protein microarray technology.

Using protein microarray analysis, we found APRIL, IL-8, MIP-1α, S100 A8/A9, and TIMP-1 in AF to be novel biomarkers for predicting SPTD at <34 weeks of gestation after the placement of rescue cerclage. APRIL, also known as tumor necrosis factor ligand superfamily member 13 (TNFSF13), is expressed by various immune cells involved in the T-helper 1 response, and regulates B-lymphocyte development and survival [21–23]. Moreover, APRIL is involved in lineage-specific regulation of placental cell viability and differentiation [24], but there is no report available on associations of APRIL with SPTD, intra-amniotic infection, and/or inflammation. In the current study, we demonstrated that AF APRIL is significantly associated with SPTD at <34 weeks of gestation after cerclage. In fact, our finding is quite evident, given the association of APRIL with Th1 profile cytokines and the association of aberrant Th1:Th2 profile (activation of the proinflammatory Th1 profile, rather than suppression of the Th2 profile) with preterm labor [25,26].

MIP-1α [chemokine ligand 3 (CCL3)] is a chemotactic chemokine involved in inflammatory and immunoregulatory processes. Elevated AF levels of MIP-1α have been reported to be associated with SPTD or intra-amniotic infection [27–29]. In asymptomatic cervical insufficiency and short cervix, regardless of cerclage placement, AF levels of MIP-1α are significantly higher in women who delivered at ≤32 weeks and <34 weeks of gestation compared with those who delivered at >32 weeks and ≥34 weeks [30,31]. This is well in agreement with the current study. Taken together, these fndings suggest that MIP-1 α is largely involved in SPTD.

S100A8/A9, also known as calprotectin, exists in the form of a heterodimer and is constitutively expressed in neutrophils and monocytes. It induces cytokine secretion and stimulates leukocyte recruitment, and is hence known to be an important modulator in inflammation [32]. Consistent with its biological characteristics, S100A8/A9 can be used as a candidate biomarker for diagnosis, prognosis, and therapeutic monitoring of inflammation-associated diseases [32]. In the context of preterm labor/preterm premature rupture of membranes, S100A8/A9 in AF or plasma is associated with histological chorioamnionitis, intra-amniotic inflammation, and shorter amniocentesis-to-delivery interval [15,33,34]. These results are in agreement with those of the current study, given reports showing associations of SPTD after cerclage for cervical insufficiency with intra-amniotic inflammation and histologic chorioamnionitis [7,18]. In the present study, S100A8/A9 showed the highest AUC (0.857, 95% confidence interval (95% CI): 0.740–0.975) amongst the five AF biomarkers. Thus, further clinical trials are required to confirm the clinical utility of this marker in identifying the best candidates for rescue cerclage.

TIMP-1 is a tissue inhibitor of matrix metalloproteinases, which play an important physiological role in remodeling the extracellular matrix, especially cervical remodeling in the obstetrics field. Increased serum TIMP-1 levels are involved in the mechanism of preterm parturition [35]. In addition, TIMP-1 in AF has been studied extensively in association with SPTD in women with preterm labor. However, in contrast with the current study, no significant association of this biomarker with SPTD has been reported [36,37]. The discrepancy between the results of the present study and other studies [36,37] may be because of the differences in the population studied (women with cervical insufficiency versus women with preterm labor; asymptomatic versus symptomatic). Our previous research on cervicovaginal fluid (CVF) in a population similar to that in the present study has also shown that TIMP-1 in CVF may be a useful predictor of SPTD at <32 weeks of gestation in women with cervical insufficiency or a short cervix [38].

There are several limitations to be considered in our study. First, this was a retrospective study with relatively small number of study participants, which thus may only provide pilot information on the role of various AF proteins in predicting cerclage outcome. Second, it was conducted in a single center, and ELISA validation was not performed on a completely independent cohort owing to limitations of sample size. These limitations could limit the generalization of our findings to other populations. Therefore, further multicenter studies are required to be performed in future to validate our findings in other cohorts. Third, during the validation work, the associations of only five of the ten (50%) proteins represented on the array-based screening experiment for SPTD at <34 weeks were reproduced by ELISA using individual samples. This discrepancy may be due to differences in the samples used for measurements (pooled versus individual samples), and the analytical characteristics of assay methods (antibody array versus ELISA). It has been reported that (i) sample pooling may not closely represent the biological average of the individual samples, and (ii) the biological variance between pools is reduced when compared with that between the individual samples [39,40]. Additionally, it has been demonstrated that ELISA can lead to false positive results, which is attributed to the cross-reactivity of the detecting antibody with other proteins or endogenous substances present in the biological samples [41,42]. Fourth, assays for AF biomarkers identified in this study are not currently available commercially, thereby limiting the clinical application of these biomarkers. Therefore, the development of point-of-care testing (also known as a rapid bedside test) for these proteins, especially S100A8/A9, in AF is required to improve the clinical application of the biomarkers for assessing pregnancy outcome after placement of the rescue cerclage. Fifth, despite our AF samples being stored at −80°C, the possibility of protein degradation events during long-term sample storage cannot be fully excluded, as suggested by a previous study [43]. Sixth, we did not perform a sample size calculation before conducting the study; the sample size was set as 20 subjects in each group, as this was the available number of patients in our hospital during the study period. It is possible that this relatively small sample size could be too underpowered (type-II error) to detect associations between AF protein levels and SPTD after rescue cerclage. Seventh, biomarker measurements using AF may be clinically unfeasible for routine use owing to their need for invasive procedures (i.e., amniocentesis). Eighth, visibility to the naked eye as a spot selection criterion tends to be subjective, which may affect the selection of target candidate proteins. The strengths of the current study were as follows: (i) it is the first biomarker study in which protein microarray technique was used to evaluate AF proteins in cervical insufficiency; (ii) it provides a comprehensive and detailed assessment of the proteins associated with SPTD at <34 weeks after cerclage; and (iii) the study design adjusted for compounding factors (e.g., cervical dilatation) in the multivariate analyses.

## Conclusion

In conclusion, using protein–antibody microarray technology, we have identified several novel biomarkers—APRIL, IL-8, MIP-1α, S100 A8/A9, and TIMP-1, which are associated with the development of SPTD at <34 weeks of gestation after rescue cerclage in the AF from women with cervical insufficiency. Large and multicenter prospective studies are further required to confirm their clinical utility in identifying potential women candidates for the placement of rescue cerclage.

## Data Availability

The data used to support the findings of the present study are included within the article. The primary data used to support the findings of the present study are available from the corresponding author upon request.

## Abbreviations

AF: amniotic fluid
APRIL: a proliferation-inducing ligand
AUC: area under the ROC curve
CV: coefficient of variation
DEP: differentially expressed protein
ELISA: enzyme-linked immunosorbent assay
IL-8: interleukin-8
MIP-1α: macrophage inflammatory protein-1α
Pref-1: preadipocyte factor 1
ROC: receiver operating characteristic
SPTD: spontaneous preterm delivery
S100 A8/A9: S100 calcium-binding protein A8/A9 complex
TIMP-1: tissue inhibitors of metalloproteinase-1
